# The Relationship between Low 25-Hydroxyvitamin D and Cardio-Metabolic Risk Factors among Ellisras Young Adults

**DOI:** 10.3390/ijerph17207626

**Published:** 2020-10-19

**Authors:** Betty Sebati, Kotsedi Monyeki, Susan Monyeki

**Affiliations:** Department of Physiology and Environmental Health, University of Limpopo, Sovenga 0727, South Africa; bettysebati800@gmail.com (B.S.); msmonyeki@yahoo.com (S.M.)

**Keywords:** 25(OH)D, cardio-metabolic, risk factor, adiposity, young adults

## Abstract

*Introduction:* 25-hydroxyvitamin D (25(OH)D) is found in circulating blood and is regarded as an estimate of vitamin D status. Low circulating 25(OH)D levels are associated with a high body mass index (BMI), increased weight and the increased development of adipose tissue. This study aimed to determine the relationship between low 25(OH)D and cardio-metabolic risk factors among Ellisras young adults. *Materials and methods:* This is a cross-sectional study that took place in a rural area at Ellisras in Limpopo Province South Africa. The study included 631 young adults (327 females and 304 males) aged between 20 and 29 years. Anthropometric measurements including height, weight and waist circumference were measured following standard procedures. Blood pressure, pulse pressure and blood parameters including fasting plasma glucose, total cholesterol and triglycerides were also measured. Correlations and linear regression were performed to determine the relationship between low 25(OH)D and cardio-metabolic risk factors. *Results:* Descriptive statistics showed significant (*p* < 0.05) mean difference of LDL, HDL and blood pressure between males and females. There was a significant association between low 25(OH)D and WC (*p* = 0.010) based on Spearman correlation. There was no association found between low 25(OH)D and HDL in all models (B ranges from 0.072 to 0.075). There was also no association found between low 25(OH)D and systolic blood pressure (SBP) in all models (B ranges from −0.009 to −0.024). *Conclusion:* Low 25(OH)D was correlated with WC, and therefore with adiposity. Knowledge of the associations between 25(OH)D deficiency and cardio-metabolic risk before the development of the disease is therefore important to establish whether 25(OH)D supplementation can be used for the prevention of these conditions. Educational programmes should be implemented to educate the communities and the nation at large on how to prevent 25(OH)D deficiency.

## 1. Introduction

Vitamin D, also referred to as 25-hydroxyvitamin D (25(OH)D), is a fat-soluble vitamin found in circulating blood and is regarded as an estimate of vitamin D status [1]. Moreover, 25(OH)D is physiologically a part of several human processes, including bone turnover and calcium homoeostasis, cardiovascular regulation, muscle and brain functions among others [2].

The deficiency of 25(OH)D has been discovered to be an independent risk factor for the potential development of cardiovascular disease risk factors [3]. Vitamin D’s mechanism on the cardiovascular system is by reducing the renin-angiotensin-aldosterone system activity, reduction in blood pressure values, and having an anti-inflammatory, anti-hypertrophic, anti-proliferative, anti-diabetic, anti-thrombotic and anti-fibrotic effects, as well as a beneficial modulation on the typical cardiovascular risk factors. Maintaining an ideal vitamin D level is vital for cardiovascular risk among other conditions and vitamin D deficiency is also considered as a cardiovascular risk marker [4]. These risk factors contribute to the development of diseases such as hypertension, diabetes, metabolic syndrome, cancer, autoimmune and infectious diseases, which are the leading causes of morbidity and mortality in the developed world [5,6].

There is still uncertainty regarding 25(OH)D prevalence due to lack of data from several countries [7]. However, Holick and Chen [7] reported that approximately 1 billion people have low 25(OH)D levels including all ethnicities and age groups. Moreover, some review studies reported a high prevalence of 25(OH)D deficiency globally [8,9,10].

Inverse associations between cardio-metabolic risk factors and 25(OH)D levels were reported in some cross-sectional studies [11,12]. Furthermore, prospective studies proposed that low levels of 25(OH)D could be useful in predicting the development of cardio-metabolic risks [13,14,15], and diseases [16,17]. However, it is maintained that adiposity intercedes the detected associations between 25(OH)D deficiency and cardio-metabolic risks, based on 25(OH)D being fat-soluble and therefore decreasing with increasing adiposity [18].

Vitamin D (25(OH)D) studies had not previously been carried out among the Ellisras population, but studies on cardiovascular and metabolic risk factors and the syndrome have been reported [19,20]. Sekgala et al. [19] found the prevalence of metabolic syndrome of 36.8% and 8.6% among Ellisras young adults’ females and males, respectively, while Sebati et al. [20] reported 1.6%, 15.7% and 64.9% prevalence of type 2 diabetes, hypercholesterolemia and dyslipidaemia, respectively.

Therefore, the aim is to determine the relationship between 25(OH)D and cardio-metabolic risk factors among Ellisras young adults.

## 2. Materials and Methods

### 2.1. Geographical Area

Ellisras is located in the rural north-westerly area of Limpopo province, South Africa. The population size is approximately 50,000, sparsely spread across 42 villages [21]. Ellisras town, which is currently called Lephalale, is situated near the Botswana border. Poverty, low-life expectancy and unemployment appear to play a substantial role in the South African rural populations, of which Ellisras community is not an exception [22,23].

#### 2.1.1. Sample

A total of 731 subjects participated in a dietary intake survey and 100 subjects refused to take part in the blood sample survey, citing cultural believes as the main reason for not taking part. They were eventually left out of the analysis. A total of 631 young adults aged 20 to 29 years (304 males with a mean age of 25.46 years, and 327 females with a mean age of 25.67 years) who were part of the Ellisras Longitudinal Study (ELS) participated in this study in November 2015. The details of ELS were explained elsewhere [24].

The Ethics Committee of the University of Limpopo granted ethical approval before the survey with ethical clearance number MREC/P/204/2013: IR. The participants signed consent forms before data collection.

Participants who were pregnant at the time of measurements, those who were hospitalised or constantly taking medication, and those with severe diseases, including cancer, were excluded. Moreover, only participants that had been part of the ELS from the onset, were healthy and available during the time of measurement participated in the study.

#### 2.1.2. Anthropometric Measurements

All participants undertook a sequence of anthropometric measurements of waist circumference (WC), weight and height following the standard procedures of the International Society for the Advancement of Kinanthropometry [25]. Trained research assistants provided the needed support during data collection. An automated scale was utilised to carry out weight measurements (to the closest 0.1 kg), while height was measured using a Martin anthropometer (to the closest 0.1 cm). Body mass index (BMI) was subsequently calculated based on the participants’ weight and height measurements. Waist circumference (WC) was measured to the nearest 0.1 cm using a retractable steel tape measure. Measurements of WC were undertaken as the participants stood upright and after a mild expiration. The anatomical landmarks used were for WC, laterally, the midpoint between the iliac crest and the bottom part of the thoracic cage, and, anteriorly, the midpoint between the xiphoid process of the navel and sternum [25].

#### 2.1.3. Blood and Pulse Pressure

Using an electronic Micronta monitoring kit, at least three readings of pulse pressure and blood pressure (BP) readings of systolic blood pressure (SBP) and diastolic pressure (DBP) were taken at an interval of five minutes apart after the participants who had been seated for 5 min or longer [26], the average from the three BP and pulse pressure readings were calculated.

#### 2.1.4. Biochemical Analysis

Participants fasted for 8–10 h before blood collection. All blood collections were done in schools by registered nurses from Witpoort Hospital in the morning. Blood samples were then placed in a cooler box with ice (2–8 °C) on-site before transported to the laboratory at the Witpoort Hospital situated in Ellisras. Fasting blood samples were centrifuged at 2500 rpm for 15 min prior to analysis and stored in a bio freezer at −80 °C for later analysis.

Fasting blood glucose (FBG) was drawn into fluoride tubes then measured using Accu-chek [27]. The total cholesterol (TC) and high-density lipoprotein cholesterol (HDL-C) levels were measured by an enzymatic (cholesterol esterase, oxidase and peroxidase) spectrophotometric technique. Low-density lipoprotein cholesterol (LDL-C) was calculated from the Friedewald equation (in mmol/L) (LDL-C = TC − HDL-C − TG/2.2), provided the triglycerides did not exceed 4.5 mmol/L. [28]. Triglycerides (TG) were measured using a standard enzymatic colourimetric method. All measurements were carried out using an AU480 Chemistry System from Beckman Coulter, while 25(OH)D was measured using access 2 Beckman Coulter immunoassay system analyser (Brea, Calif).

The instruments were calibrated according to standard procedures. All biochemical analyses were carried out by the staff members of the Medical Science Unit of the Department of Pathology and Medical Science at the University of Limpopo.

#### 2.1.5. Statistical Analysis

All data analyses were prepared using SPSS version 23 (IBM, Chicago, Ill, USA), *p*-value was set at *p* ≤ 0.05. Descriptive statistics were done for all the variables (mean and standard deviation for normally distributed variables and median and interquartile range for non-normally distributed variables designated by 3 on Table 1). A Student test was used to compare the means between the normally distributed variables, while a non-parametric T-test was used for the variables that were not normally distributed. Partial and Spearman correlations were performed to determine the association between low 25(OH)D and cardo-metabolic risk factors. Partial correlation was controlled for age and sex separately. Linear regression was performed to determine the association between low 25(OH)D and cardio-metabolic risk factors adjusted for age, sex, BMI and WC.

## 3. Results

### 3.1. Characteristics of the Sample Size

Table 1 shows descriptive statistics (mean and standard deviation for normally distributed variables and median and interquartile range for non-normally distributed variables) for the anthropometric and cardio-metabolic risk factors of the study sample. Males showed a higher mean of SBP, DBP and HDL (125.89, 71.39 and 1.20, respectively) than females (114.13, 69.04 and 1.09, respectively), while females showed a higher mean BMI and WC (24.91 and 82.20, respectively) than the males (24.77 and 75.07, respectively). There was a significant (*p* < 0.05) mean difference in LDL, HDL and blood pressure between males and females. There was also significant mean difference in TC/HDL and LDL/HDL, while TG/HDL showed a non-significant difference between males and females.

### 3.2. The Relationship between Low 25(OH)D and Cardio-Metabolic Risk Factors by Correlation

Table 2 shows the correlation between low 25(OH)D and cardio-metabolic risk factors. There was a significant association between low 25(OH)D and WC (*p* = 0.010) in Spearman correlations only. There was no association between low 25(OH)D and SBP (*p* = 0.682–0.875) uncontrolled and controlled for age and sex. There was no significant association found between low 25(OH)D and TC/HDL, TG/HDL and LDL/HDL ratios (*p* value = 0.073 to 0.785) in both Spearman and Partial correlations.

### 3.3. The Relationship between Low 25(OH)D and Cardio-Metabolic Risk Factors by Linear Regression

Table 3 shows linear regression between low 25(OH)D and cardio-metabolic risk factors adjusted for age, sex, BMI and WC. There was no association found between low 25(OH)D and HDL in all models (B ranges from 0.072 to 0.075). There was also no association found between low 25(OH)D and SBP in all models (B ranges from −0.009 to −0.024). Furthermore, there was no significant association between low 25(OH)D and all the ratios, namely TC/HDL, TG/HDL and LDL/HDL (B ranges from −0.15 to −0.05) in all models.

## 4. Discussion

This study aimed to determine the relationship between low 25(OH)D and cardio-metabolic risk factors among Ellisras young adults. A significant association was found between WC (as an indicator of adiposity) and low 25(OH)D, while there was no association between low 25(OH)D and BMI, LDL, HDL, total cholesterol, triglycerides, fasting glucose, TG/HDL, TC/HDL, LDL/HDL SBP, DBP and pulse pressure among Ellisras young adults. The non-significant association between 25(OH)D and HDL found in the current study could partly be responsible for the non-significant associations between 25(OH)D and all the TG/HDL, TC/HDL and LDL/HDL ratios since all of them have HDL as their denominator. Numerous studies have reported inverse associations between 25(OH)D and glucose (both fasting and 2 h), some of which had more than 10,000 participants [29,30,31,32,33]. The current study did not find any association between low 25(OH)D and fasting glucose. One of the reasons behind the findings of the current study and the above-mentioned studies could be due to the big difference in the sample size (the current study had a smaller sample size). Although the current study did not find any association between vitamin D (25(OH)D) and blood pressure, it has been reported that low vitamin D status is associated with an elevated prevalence of hypertension [34]. Furthermore, it was revealed that significantly increased rates of hyperlipidaemia, diabetes and peripheral vascular diseases were observed in people with low levels of 25(OH)D [35].

A study by Mousa et al. [3] reported that low 25(OH)D levels in circulation were linked to increased cardio-metabolic risk factors such as adiposity degree, inflammation, glucose intolerance and insulin resistance in univariate analyses. Nonetheless, the associations between 25(OH)D and the above-mentioned risk factors (excluding pulse pressure and fasting glucose) were not significant when adjusted for percentage body fat. The current study also found a significant association between WC (which reflects adiposity in the abdominal area) and low 25(OH)D. This could suggest that the associations between 25(OH)D deficiency and cardio-metabolic risks are influenced by adiposity [3]. Moreover, abdominal obesity has been related with increased cardiovascular risks, hyperinsulinemia, heart disease and high blood pressure, among others [36], and that might affect plasma/circulating 25(OH)D level.

Vitamin D deficiency was found to be prevalent in 30–50% of the general population, and prevalence approximation suggests that greater than 1 billion people are vitamin D-deficient globally [35]. The current study found the prevalence of 25(OH)D deficiency to be 96.6% based on the <12 ng/mL (<30 nmol/L) cut off point and 1.9% using the <20 ng/mL cut off point (<50 nmol/L) [37], while Wang et al. [38] reported 25(OH)D deficiency prevalence of 72% based using the <50 nmol/L D cut off point and 6.6% prevalence based on <25 nmol/L among young adults aged 18–26 in Hong Kong. Moreover, Shifinaz and Moy [37] also reported a high prevalence of 25(OH)D deficiency among teachers in Malaysia. The higher prevalence of 25(OH)D in the current study could be due to the dark skin pigmentation of the participants (they are all black). The high content of melanin in dark skin prevents the synthesis of 25(OH)D [39]. Moreover, Tsiaras and Weinstock [40] reported that people with darker skin pigmentation or a higher content of melanin needed a lengthier sun exposure than people with lighter skin to produce a similar amount of 25(OH)D [40].

There were several limitations to the current study. The sample size was small. The measure/reflector of adiposity/body fat was not the most accurate and sensitive measure of adiposity. Moreover, there are numerous procedures of determining plasma 25(OH)D and these may influence the readings of 25(OH)D level. The possibility of bias cannot be excluded from the study.

## 5. Conclusions

The prevalence of 25(OH)D deficiency was high and poorly associated with the majority of the cardio-metabolic risk factors. Low 25(OH)D was correlated with WC, and therefore with adiposity. Knowledge of the associations between 25(OH)D deficiency and cardio-metabolic risk before the development of the disease is therefore important to establish if 25(OH)D supplementation can be used for the prevention of these conditions. Educational programmes should be implemented to educate the communities and the nation at large on how to prevent 25(OH)D deficiency.

## Figures and Tables

**Table 1:** Characteristics of the study sample by sex.

		All	Males (*N* = 304)	Females (*N* = 327)	
Variables	*N*	M(SD)	M(SD)	M(SD)	*p*-Value
Age (years)	631	25.57 (1.98)	25.46 (1.93)	25.67 (2.03)	0.297
25(OH)D ^#^ (ng/mL)	631	0.43 (0.00–1.43)	0.46 (0.00 1.41)	0.43 (0.00–1.44)	0.286
LDL (mmol/L)	631	1.99 (0.92)	1.77 (0.87)	2.22 (0.91)	0.000 *
HDL (mmol/L)	631	1.15 (0.34)	1.20 (0.37)	1.09 (0.30)	0.000 *
Total cholesterol (mmol/L)	631	4.15 (1.04)	4.03 (0.94)	4.27 (1.11)	0.004 *
Triglyceride ^#^ (mmol/L)	631	0.83 (0.63–1.19)	0.87 (0.65–1.22)	0.78 (0.61–1.18)	0.028 *
Fasting blood glucose (mmol/L)	631	5.52 (1.28)	5.59 (0.88)	5.43 (1.56)	0.165
SBP (mmHg)	631	119.83 (13.06)	125.89 (10.17)	114.13 (10.85)	0.000 *
DBP (mmHg)	631	70.18 (9.81)	71.39 (10.17)	69.04 (9.32)	0.001 *
Pulse pressure (mmHg)	631	76.11 (13.38)	71.39 (12.83)	81.29 (11.76)	0.000 *
BMI (kg/m^2^)	631	24.62 (13.15)	24.77 (12.22)	24.91 (15.43)	0.000 *
WC (cm)	631	78.74 (12.75)	75.07 (9.50)	82.20 (14.36)	0.000 *
**Ratios**					
TG/HDL	631	0.79 (0.54–1.10)	0.78 (0.52–1.08)	0.80 (0.55–1.13)	0.405
TC/HDL	631	3.80 (1.19)	3.55 (1.27)	4.03 1.05)	0.000 *
LDL/HDL	631	2.61 (1.14)	2.36 (1.22)	2.84 (1.00)	0.000 *
**The prevalence of 25(OH)D status**					
**Cut off point**			***N*(%)**		
<12 ng/mL			706 (96.6)		

Mean (standard deviation); 25(OH)D: 25-Hydroxyvitamin D; LDL: low-density lipoprotein cholesterol; HDL: high-density lipoprotein cholesterol; SBP: systolic blood pressure; DBP: diastolic blood pressure; BMI: body mass index; WC: waist circumference; ng/mL: nanograms/millilitre; mmol/L: millimoles/litre; mmHg: millimetres of mercury; ^#^ = median and 25 and 75 interquartile), * *p* ≤ 0.05.

**Table 2:** Correlation (Spearman and partial) between low 25(OH)D and cardio-metabolic risk factors.

Variables	Spearman Correlation Coefficients (*p*-Value)	Partial Correlation Coefficients ^1^ (*p*-Value) [AGE]	Partial Correlation Coefficients ^2^ (*p*-Value) [SEX]
LDL (mmol/L)	0.042 (0.298)	0.043 (0.374)	0.052 (0.291)
HDL (mmol/L)	0.050 (0.206)	0.078 (0.110)	0.073 (0.135)
Total cholesterol (mmol/L)	0.054 (0.179)	0.065 (0.183)	0.069 (0.156)
Triglyceride (mmol/L)	0.12 (0.062)	0.01 (0.839)	0.01 (0.841)
Fasting blood glucose (mmol/L)	0.015 (0.696)	0.010 (0.836)	0.012 (0.798)
SBP (mmHg)	−0.015 (0.682)	0.020 (0.689)	0.008 (0.875)
DBP (mmHg)	0.067 (0.070)	0.031 (0.528)	0.027 (0.580)
Pulse pressure (mmHg)	0.026 (0.477)	−0.027 (0.578)	−0.016 (0.742)
BMI (kg/m^2^)	0.022 (0.546)	0.050 (0.307)	0.058 (0.236)
WC (cm)	0.096 (0.010) *	0.053 (0.277)	0.059 (0.230)
**Ratios**			
TG/HDL	0.03 (0.472)	−0.02 (0.562)	−0.04 (0.370)
TC/HDL	−0.07 (0.073)	−0.01 (0.785)	−0.01 (0.736)
LDL/HDL	−0.03 (0.489)	−0.02 (0.566)	−0.04 (0.368)

Coefficients ^1^: controlled for age; Coefficient ^2^: controlled for sex; LDL: low-density lipoprotein cholesterol; HDL: high-density lipoprotein cholesterol; SBP: systolic blood pressure; DBP: diastolic blood pressure; BMI: body mass index; WC: waist circumference; mmol/L: millimoles/litre; mmHg: millimetres of mercury *p* ≤ 0.05.

**Table 3:** Linear regression for the relationship between low 25(OH)D and cardio-metabolic risk factors.

	Model 1		Model 2		Model 3		Model 4	
Dependent Variable	B (95% CI)	*p*-Value	B (95% CI)	*p*-Value	B (95% CI)	*p*-Value	B (95% CI)	*p*-Value
**LDL (mmol/L)**	0.059 (−0.045–0.215)	0.201	0.051 (0.057–0.201)	0.272	0.051 (−0.057–0.202)	0.273	0.049 (0.0059–0.199)	0.287
**HDL (mmol/L)**	0.072 (−0.011–0.084)	0.129	0.074 (−0.004–0.031)	0.121	0.075 (−0.003–0.032)	0.114	0.075 (−0.003–0.032)	0.114
**Total cholesterol (mmol/L)**	0.074 (−0.032–0.270)	0.121	0.062 (−0.048–0.248)	0.186	0.058 (−0.053–0.242)	0.209	0.057 (−0.055–0.239)	0.220
**Triglyceride (mmol/L)**	0.005 (−0.031–0.035)	0.918	−0.011 (−0.036–0.028)	0.820	−0.011 (−0.035–0.028)	0.829	−0.004 (−0.034–0.031)	0.931
**Fasting blood glucose (mmol/L)**	0.031 (−0.007–0.015)	0.503	0.025 (−0.008–0.014)	0.583	0.025 (−0.008–0.014)	0.584	0.024 (−0.008–0.014)	0.598
**SBP (mmHg)**	−0.009 (−1.836–1.445)	0.815	−0.019 (−1.994–1.212)	0.632	−0.023 (−2.064–1.111)	0.556	−0.024 (−2.082–1.091)	0.540
**DBP (mmHg)**	0.027 (−0.943–1.795)	0.541	0.023 (−0.990–1.729)	0.594	0.020 (−1.039–1.669)	0.648	0.020 (−1.044–1.666)	0.652
**Pulse pressure (mmHg)**	0.013 (−1.411–1.981)	0.732	0.016 (−1.354–2.041)	0.691	0.011 (−1.454–1.937)	0.779	0.013 (−1.420–1.964)	0.752
**Ratios**								
**TC/HDL**	−0.10 (−0.33–0.13)	0.414	−0.10 (−0.33–0.13)	0.414	−0.15 (−0.38 0.09)	0.224	−0.15 (−0.38–0.09)	0.224
**TG/HDL**	−0.05(−0.48–0.38)	0.820	−0.05 (−0.48–0.38)	0.819	−0.14 (−0.58– 0.30)	0.521	−0.14 (−0.58–0.30)	0.521
**LDL/HDL**	−0.10(−0.34–0.14)	0.408	−0.10 (−0.34–0.14)	0.407	−0.15 (−0.39–0.10)	0.232	−0.15 (−0.39–0.10)	0.232

B: beta; CI: confidence interval; Model 1: 25(OH)D, age and sex; Model 2: Model 1 + BMI; Model 3: Model 1 + WC; Model 4: Model 1 + BMI + WC; LDL: low-density lipoprotein cholesterol; HDL: high-density lipoprotein cholesterol; SBP: systolic blood pressure; DBP: diastolic blood pressure; BMI: body mass index; WC: waist circumference; mmol/L: millimoles/litre; mmHg: millimetres of mercury; *p* ≤ 0.05.
